# Cost-effectiveness of implementing risk-based cardiovascular disease (CVD) management using updated WHO CVD risk prediction charts in India

**DOI:** 10.1371/journal.pone.0285542

**Published:** 2023-08-25

**Authors:** Parthibane Sivanantham, Mathan Kumar S., Saravanan Essakky, Malkeet Singh, Srobana Ghosh, Abha Mehndiratta, Sitanshu Sekhar Kar

**Affiliations:** 1 Department of Preventive and Social Medicine, Jawaharlal Institute of Postgraduate Medical Education and Research (JIPMER), Puducherry, India; 2 Center for Global Development, Europe; Parul University, INDIA

## Abstract

**Introduction:**

The World Health Organization (WHO) has released the updated cardiovascular disease (CVD) risk prediction charts in 2019 for each of the 21 Global Burden of Disease regions. The WHO advocates countries to implement population-based CVD risk assessment and management using these updated charts for preventing and controlling CVDs.

**Objective:**

To assess the cost-effectiveness of implementing risk-based CVD management using updated WHO CVD risk prediction charts in India

**Methods:**

We developed a decision tree combined with Markov Model to simulate implementing two community-based CVD risk screening strategies (interventions) compared with the current no-screening scenario. In the first strategy, the whole population is initially screened using the WHO non-lab-based CVD risk assessment method, and those with ≥10% CVD risk are subjected to WHO lab-based CVD risk assessment (two-stage screening). In the second strategy, the whole population is subjected only to the lab-based CVD risk assessment (single-stage screening). A mathematical cohort of those aged ≥40 years with no history of CVD events was simulated over a lifetime horizon with three months of cycle length. Data for the model were derived from a primary study and secondary sources. Incremental cost-effectiveness ratios (ICERs) were determined for the screening strategies and sensitivity analyses.

**Results:**

The discounted Incremental cost-effectiveness ratio per QALY gained for both the two-stage (US$ 105; ₹ 8,656) and single-stage (US$ 1073; ₹ 88,588) screening strategies were cost-effective at an implementation effect of 40% when compared with no screening scenario. Implementing CVD screening strategies are estimated to cause substantial reduction in the number of CVD events in the population compared to the no screening scenario.

**Conclusion:**

In India, both CVD screening strategies would be cost-effective, and implementing the two-staged screening would be more cost-effective. Our findings support implementing population-based CVD screening in India. Future studies shall assess the budget impact of these strategies at different implementation coverage levels.

## Introduction

Globally, cardiovascular diseases (CVD) account for two-fifth (42%) of all deaths caused due to non-communicable diseases (NCD) [1]. Nearly half (52%) of all premature deaths worldwide are due to CVDs. Each year, CVDs cause about 17.5 million deaths worldwide [2]. Of this, more than 80% occur in the low and middle-income countries (LMICs) [1]. Between 1990 and 2013, South Asian countries witnessed the most significant increase (97.4%) in CVD-associated deaths [3]. In India, about one in four deaths are due to CVDs, mainly ischemic heart disease and stroke [4].

To tackle NCD-associated morbidity and mortality, the UN General assembly and its member states have adopted a global target to achieve a 25% relative reduction in premature deaths due to NCDs by 2025 [5]. The NCD global monitoring framework has also aimed to reach at least 50% of the population having a high total CVD risk (≥ 30% or a history of CVD events) with drug treatments and counselling to prevent CVD events (heart attack and stroke) in the population. In line with this, India has adopted the above-stated global targets to reduce premature mortality from CVDs among the high CVD risk population in the country [6, 7]. The World Health Organization (WHO) also advocates governments initiate drug and lifestyle management for those having ≥20% total CVD risk as one of the ‘best-buy’ strategies in the prevention and control of NCDs [8].

Several CVD risk prediction tools have been developed over the years and across countries, including the recent ‘updated WHO CVD risk prediction charts’ (WHO HEARTS Package) developed for each of the 21 global regions [7, 9–11]. The charts have two versions; non-lab-based CVD risk assessment is recommended for low resource settings, and the lab-based assessment is recommended for setting where resources are available [7, 10, 12].

WHO has also been advocating and assisting countries in implementing population-based CVD risk assessment and management using the risk-based CVD management module of the WHO HEARTS package [13, 14]. For LMICs like India, where there are resource constraints, it is not sufficient that a public health intervention is effective; but also needs to be cost-effective for considering implementing the intervention. In recent years, although countries have implemented both the lab and non-lab-based CVD risk assessments prescribed by the HEARTS package, the cost-effectiveness of implementing the HEARTS-based CVD risk assessment has not been evaluated yet [13, 14].

HEARTS-based CVD risk assessment is not currently implemented at the population level in India. Given the high burden of CVD in the country, assessing the cost-effectiveness of implementing risk-based CVD management using updated WHO CVD risk prediction charts can provide valuable information for policy decisions regarding the prevention and control of CVDs. Therefore, we have undertaken an economic evaluation of HEARTS-based CVD risk assessment in India to inform policy decisions on implementing this public health intervention.

## Methodology

We used the decision tree and Markov model to evaluate the cost-effectiveness of implementing the WHO-HEARTS technical package for CVD risk assessment in India by comparing it with the no screening scenario. Ethics approval was not required as the model was developed using data from secondary sources.

### No screening scenario (comparator)

We compared the intervention with the no screening scenario as currently there is no population-based screening for CVD risk assessment in India.

### Screening strategies (intervention)

**Fig 1:**
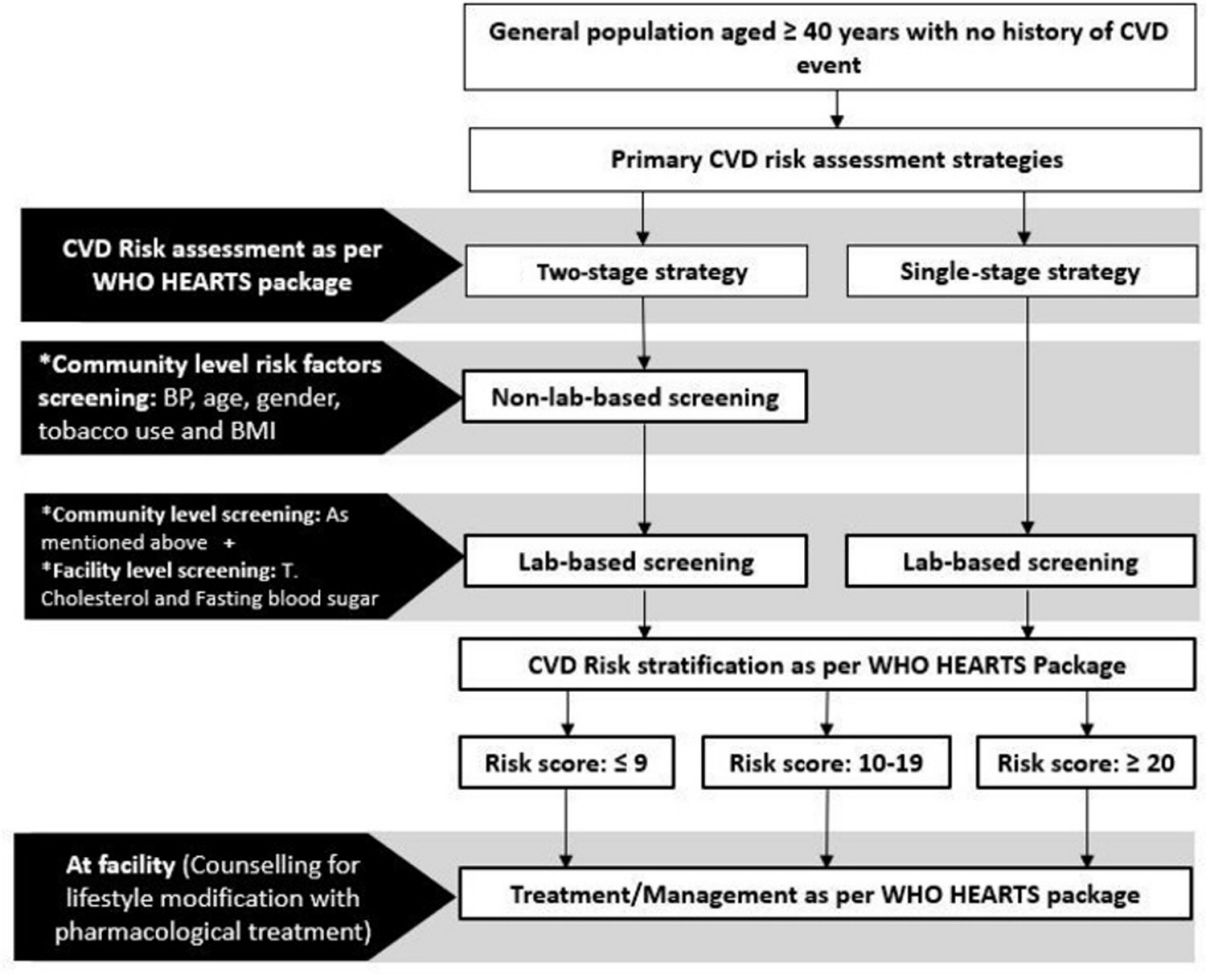
CVD risk assessment strategies (two-stage and single-stage screening). * Risk factors screening done as per WHO STEPS guidelines for NCR risk factors assessment.

We considered two screening strategies (Fig 1). In the first strategy, the whole population is initially screened using non-lab-based CVD risk assessment method, and those with ≥10% CVD risk are subjected to lab-based CVD risk assessment (two-stage screening). The whole population is subjected to the lab-based CVD risk assessment (single-stage screening) in the second strategy. Currently, WHO recommends two-stage CVD risk assessment in resource-poor settings where it is not feasible to subject the whole population to lab-based risk assessment. Single-stage CVD risk assessment is recommended for settings where resources for laboratory estimation of CVD risk for the entire population are affordable [7]. The advantage of single-stage lab-based risk assessment is that it captures the false-negative individuals who would be missed out in the non-lab-based screening, when a two-stage screening strategy is adopted.

### Model overview and cost-effectiveness analysis

**Fig 2:**
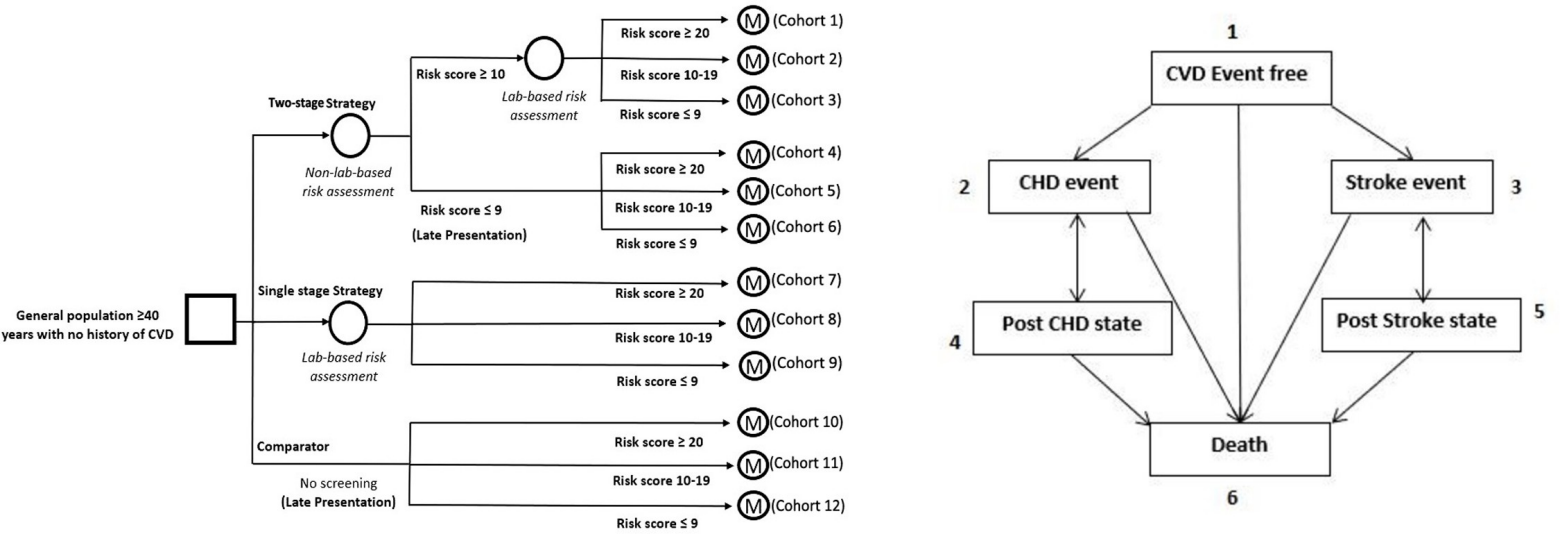
Schematic representation of Decision tree (a) and Markov model (b).

A schematic representation of the decision tree and Markov model is in Fig 2. The initial cohort in the two-stage screening strategy was segregated into different CVD risk levels based on the total CVD risk estimated using non-lab-based chart followed by the lab-based chart. Whereas the cohort in the single-stage strategy was segregated based on the CVD risk estimated from the lab-based CVD risk assessment chart (Fig 2A). We applied the incidences of stroke and coronary heart disease (CHD) in each segregated cohort as per the mean total CVD risk (%) of individuals of the respective cohort, which eventually entered into the Markov model (Fig 2B).

We developed a Markov model as per the natural history of stroke and CHD events, with five mutually exclusive health states and death as an absorbing state. The occurrence of stroke and CHD events were simulated for over 50 years with a cycle length of three months using transition probabilities given in the Table 1.

**Table 1:** Model input parameters.

Parameters	Base case	Lower limit	Upper limit	Distribution	Reference
**Epidemiological parameters**					
Probability of test positive using non-lab-based CVD risk assessment	0.148	0.178	0.118	Beta	WHO STEPS Survey
Probability of population in non-lab-based 10–20% CVD risk have lab-based > = 20% CVD risk (cohort 1)	0.190	0.228	0.152	Beta	WHO STEPS Survey
Probability of population in non-lab-based 10–20% CVD risk have lab-based 10–20% CVD risk (cohort 2)	0.705	0.846	0.564	Beta	WHO STEPS Survey
Probability of population in non-lab-based 10–20% CVD risk have lab-based < = 10% CVD risk (cohort 3)	0.105	0.126	0.084	Beta	WHO STEPS Survey
Probability of population in non-lab-based < = 10% CVD risk has lab-based > = 20% CVD risk (cohort 4)	0.002	0.002	0.002	Beta	WHO STEPS Survey
Probability of population in non-lab-based < = 10% CVD risk has lab-based 10–20% CVD risk (cohort 5)	0.100	0.120	0.080	Beta	WHO STEPS Survey
Probability of population in non-lab-based < = 10% CVD risk has lab-based < = 10% CVD risk (cohort 6)	0.898	1.078	0.718	Beta	WHO STEPS Survey
Probability of population in lab-based test having > = 20% CVD risk (cohort 7 & 10)	0.030	0.036	0.024	Beta	WHO STEPS Survey
Probability of population in lab-based 10–20% CVD risk (cohort 8 & 11)	0.190	0.228	0.152	Beta	WHO STEPS Survey
Probability of population in lab-based < = 10% CVD risk (cohort 9 & 12)	0.780	0.936	0.624	Beta	WHO STEPS Survey
Three monthly probability of CVD event from event free state (cohort 1) (≥20%)	0.006	0.007	0.005	Log-Normal	WHO STEPS Survey
Three monthly probability of CVD event from event free state (cohort 2) (10–19%)	0.004	0.004	0.003	Log-Normal	WHO STEPS Survey
Three monthly probability of CVD event from event free state (cohort 3) (1–9%)	0.002	0.003	0.002	Log-Normal	WHO STEPS Survey
Three monthly probability of CVD event from event free state (cohort 4) (≥20%)	0.006	0.007	0.004	Log-Normal	WHO STEPS Survey
Three monthly probability of CVD event from event free state (cohort 5) (10–19%)	0.003	0.004	0.003	Log-Normal	WHO STEPS Survey
Three monthly probability of CVD event from event free state (cohort 6) (1–9%)	0.001	0.001	0.001	Log-Normal	WHO STEPS Survey
Three monthly probability of CVD event from event free state (cohort 7 & 10) (≥20%)	0.006	0.007	0.005	Log-Normal	WHO STEPS Survey
Three monthly probability of CVD event from event free state (cohort 8 & 11) (10–19%)	0.003	0.004	0.003	Log-Normal	WHO STEPS Survey
Three monthly probability of CVD event from event free state (cohort 9 & 12) (1–9%)	0.002	0.002	0.001	Log-Normal	WHO STEPS Survey
Probability of CHD event	0.522	0.626	0.417	Beta	Based on Hemanshu Das et al. 2021 [17]
Probability of stroke event	0.479	0.574	0.383	Beta	
Three monthly probability of death from CHD event state	0.018	0.022	0.014	Log-Normal	
Three monthly probability of death from stroke event state	0.033	0.040	0.027	Log-Normal	
Three monthly probability of death from post CHD state	0.003	0.003	0.002	Log-Normal	
Three monthly probability of death from post Stroke state	0.003	0.003	0.002	Log-Normal	
Three monthly probability of recurrent CHD event	0.021	0.025	0.016	Log-Normal	Based on Steg et al. 2007 [18]
Three monthly probability of recurrent stroke event	0.004	0.005	0.003	Log-Normal	Petty et al. 1998 [19]
**Cost parameters (US$)**					
Treatment cost for Stroke (In-patient cost)	571	573	287	Gamma	NSSO 2017–18 [20]
Treatment cost for post Stroke (Out-patient cost i.e. follow up cost)	35	30	61	Gamma	NSSO 2017–18 [20]
Treatment cost for post CHD (Out-patient cost i.e. follow up cost)	36	42	24	Gamma	NSSO 2017–18 [20]
Treatment cost for DM (Out-patient cost i.e. follow up cost)	18	23	8	Gamma	NSSO 2017–18 [20]
Treatment cost for HTN (Out-patient cost i.e. follow up cost)	14	17	8	Gamma	NSSO 2017–18 [20]
Treatment cost for DM + HTN (Out-patient cost i.e. follow up cost)	31	39	16	Gamma	Calculated from NSSO 2017–18 [20]
Total cost of PCI	1574	1889	1259	Gamma	DHR HTA report [21]
Total cost of CABG	1591	1909	1273	Gamma	DHR HTA report [21]
Total cost of Optimal Medical therapy	178	213	142	Gamma	DHR HTA report [22]
Proportion of PCI	0.07	0.090	0.060	Beta	Expert opinion
Proportion of CABG	0.03	0.035	0.023	Beta	Expert opinion
Proportion of OMT	0.9	1.000	0.717	Beta	Expert opinion
Screening (Non-lab chart)	0.2	0.25	0.17	Gamma	Calculated
Screening (lab chart)	7	8	5	Gamma	Calculated
Intervention costs for HEARTS risk categories <9 & 10–19	0.5	0.6	0.4	Gamma	Calculated
Cost of Statin therapy (Intervention costs for HEARTS risk categories (> = 20))	1.8	2	1.4	Gamma	Calculated
**Utility parameters**					
Remains event free	1.000	1.000	0.800	Beta	HTA report (DM & HTN) [23]
Diabetes Mellitus	0.760	0.912	0.608	Beta	HTA report (DM & HTN) [23]
Hypertension	0.890	1.000	0.712	Beta	HTA report (DM & HTN) [23]
Diabetes Mellitus & HTN	0.825	0.990	0.660	Beta	Calculated
Stroke event	0.180	0.216	0.144	Beta	Pukas JD et al. [24]
CHD event	0.700	0.840	0.560	Beta	Pukas JD et al. [24]
Post CHD	0.876	1.000	0.701	Beta	Pukas JD et al. [24]
Post Stroke (non-disabling)	0.640	0.768	0.512	Beta	Expert opinion
Post Stroke (disabling)	0.180	0.216	0.144	Beta	Expert opinion
Proportion non disabling stroke	0.600	0.720	0.480	Beta	Expert opinion
Proportion disabling stroke	0.400	0.480	0.320	Beta	Expert opinion

PCI: Percutaneous Coronary Intervention; CABG: Coronary Artery Bypass Graft, OMT: Optimal Medical Therapy.

Cohorts in the intervention arms were applied with the benefits of respective treatment given for the management of high CVD risk prescribed in the WHO HEARTS risk-based CVD management module for the prevention of stroke and CHD events [7]. The cohort in the comparator arm, i.e., no screening scenario, and the cohort that tested negative for non-lab-based screening (total CVD risk of ≤9) in the two-stage strategy were also segregated into different CVD risk levels based on the total CVD risk estimated using the lab-based charts. These cohorts were also applied with the respective risk of developing CVDs that would emerge as the late presentation.

We applied the model’s cost of screening, treatment and management to the respective cohorts in the various health states. Similarly, health gain or decrement in each health state was quantified in terms of quality adjusted life years (QALY) by incorporating EQ-5D-5L-based utility scores. The model was developed from the societal perspective by combining direct and indirect medical costs incurred by patients and the health system. The cost and health utilities were discounted at the rate of 3%. The differences in the total cost, total QALY, and total life years between the intervention strategies (single or two-stage screening) and comparator were used to estimate the ICERs for the single and two-stage screening strategies. We assumed the one-time GDP per capita of India as the cost-effectiveness threshold. We have also used India’s one-time GDP per capita to monetize the health benefit and estimated net monetary benefit (NMB).

### Model inputs parameters

Input parameters used for the model are presented in Table 1. Epidemiological data related to the ‘total CVD risk’ and the prevalence of diabetes mellitus (DM), Hypertension (HTN), DM combined with HTN were obtained from a primary study conducted using WHO STEPS methodology in the district of Puducherry. Of the total participants of the survey (N = 2415), the information required for estimating the ‘total CVD risk’ using the lab and non-lab-based CVD risk prediction charts was available for 710 participants. Therefore, the required information for the current study on the proportion of population at different CVD risk levels using two-stage and single-stage CVD risk assessment, proportion of population having DM, HTN, DM & HTN was estimated from this population. The detailed information about the survey and the methodology followed for total CVD risk estimation have been published elsewhere [15, 16].

For the study, the risk and incidence of stroke and CHD were derived from the total CVD risk estimated from the CVD risk prediction charts. The ten-year probability of getting a CVD event was converted to an annual probability which was then converted into three months probability using the formula (1-((1-annual probability)^(1/12)))*3. The cost was obtained from secondary sources, and utility data was obtained from either secondary sources or expert opinion when reliable estimates were lacking.

### Sensitivity analysis

Uncertainty in the model parameter values were assessed through one-way sensitivity analysis (OWSA). OWSA was carried out in MS excel with a ±20% change in the base case values, as shown in Table 1. The results of OSWA were presented using tornado graph.

### Scenario analysis

We conducted scenario analysis by varying the clinical effectiveness of the WHO HEARTS high CVD risk management that is implemented following the CVD risk estimation in an individual. Under the prescribed high CVD risk management protocol, we assumed a range of four (0.2, 0.4, 0.6 and 0.8) cumulative relative risks of developing stroke and CHD.

## Results

### Population characteristics

Among 710 survey participants, majority (415, 58%) were women. The participants’ mean (SD) age was 53.5 (8.4) years. The prevalence of key risk factors of CVDs among the survey participants are presented in the Table 2.

**Table 2:** Baseline characteristics of WHO STEPS survey participants satisfied the criteria for CVD risk estimation for the study (N = 710).

Variables	Male (n = 295) n (%)	Female (n = 415) n (%)	Both Gender (n = 710) n (%)
Current smoking	55 (18.6)	0	55 (7.7)
Overweight^†^	65 (22)	58 (14)	123 (17.3)
Obesity	122 (41.4)	240 (57.8)	362 (51)
Raised systolic blood pressure^$^	67 (22.7)	80 (19.3)	147 (20.7)
Hypertension	139 (47.1)	173 (41.7)	312 (43.9)
Diabetes mellitus	116 (39.3)	119 (28.7)	235 (33.1)
Hypercholesterolemia	110 (37.3)	178 (42.9)	288 (40.6)

^**†**^Overweight: BMI of 23–24.99 kg/m^2^; obesity: BMI of ≥25 kg/m^2^.

^$^Raised systolic blood pressure: SBP ≥140mm Hg.

The baseline CVD risk of the study population was assessed using both the CVD screening strategies (single & two-stage). When assessed using two-stage screening strategy, 105 (14.8%, 95% CI: 12.1–17.5) of population were found to have ≥10% CVD risk in the non-lab-based screening who were subjected to the lab-based screening. In this stage, about 20 (2.8%, 95% CI: 1.5–4.2), 74 (10.4%, 95% CI: 8.3–12.7) and 11 (1.6%, 95% CI: 0.7–2.5) of the population were having ≥20%, 10–19% and ≤9% CVD risk of getting a fatal/non-fatal CVD event over the next ten years respectively. The corresponding mean (SD) CVD risk of the CVD risk groups at the end of two-stage screening were 21.85 (2), 13.91 (3) and 8.45 (0.5) respectively.

Similarly, when the baseline CVD risk was assessed using a single-stage CVD screening strategy, about 21 (3%, 95% CI: 1.8–4.2), 135 (19%, 95% CI: 16–22.6) and 554 (78%, 95% CI: 74.5–80.7) of the population were having ≥20%, 10–19% and ≤9% CVD risk respectively. The corresponding mean (SD) CVD risk of the risk groups were 20.92 (2), 13.02 (2.7) and 6.45 (2.3) respectively.

### Base case results

We found that both the two-stage and single-stage CVD screening strategies were cost-effective compared to the no screening scenario, as observed from the discounted estimates of ICER/QALY gained for the screening strategies. Among the strategies, the two-stage screening was more cost-effective than the single-stage screening (Table 3).

**Table 3:** Base case results of the model when considering the effect size of HEARTS intervention as 0.6 in CVD screening strategies (N = 100).

	No screening	*Two-stage screening	^#^Single-stage screening
Discounted estimates			
Total cost in US$ (₹)	66,809 (55,18,285)	67,818 (56,01,655)	83,036 (68,58,627)
Total QALY	1277.9	1287.5	1293.0
Total life years saved	7874	7897	7911
Incremental cost in US$ (₹)	-	1,009 (83,370)	16,227 (13,40,342)
Incremental QALY	-	9.6	15.1
Incremental life years saved	-	23	36
ICER/QALY gained in US$ (₹)	-	105 (8,656)	1,073 (88,588)
ICER/Life year saved in US$ (₹)	-	13 (1,076)	583 (48,169)

*Population is screened initially using non-lab-based CVD risk assessment method, and those having ≥10% CVD risk were subjected to lab-based CVD risk assessment

^**#**^Population is screened only using the lab-based CVD risk assessment method

The discounted ICERs per life-years saved, incremental QALY gained, incremental life-years saved, total cost incurred, and QALY gained for both the screening strategies are presented in Table 3.

Net Monitory Benefit (NMB) associated with both the strategies are presented in Table 4. Assuming the threshold value (λ) as one-time GDP per capita income of India (US$ 2,055; ₹ 1,69,625 in 2021–22), the NMB for the two-stage and single-stage screening were US$ 188 (₹ 15,503) and US$ 148 (₹ 12,261), respectively, for an individual (N = 1).

**Table 4:** Net monetary benefit associated with screening with two-stage and single-stage CVD risk assessment strategies (N = 1).

Screening strategies	Incremental cost US$ (₹)	Incremental benefit US$ (₹)	Net monetary benefit US$ (₹)
**Two-stage screening**	10 (834)	198 (16,337)	188 (15,503)
**Single-stage screening**	162 (13,403)	311 (25,664)	148 (12,261)

### One-way sensitivity and scenario analysis

**Fig 3:**
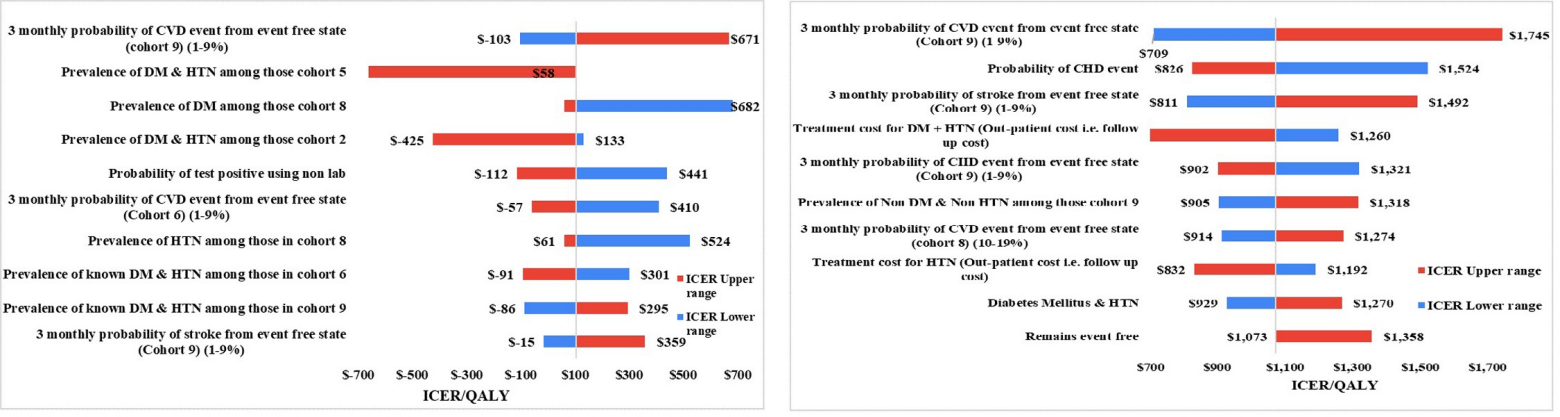
**a:** One-way sensitivity analysis for two-stage CVD screening strategy. **b:** One-way sensitivity analysis for Single-stage CVD screening strategy.

In the two-stage screening, the parameters that had most influence on the ICER were ‘Three monthly probability of CVD event from event free state of Cohort 9’, ‘Prevalence of DM & HTN among those in the CVD risk cohort 5’ and ‘Prevalence of DM among those in cohort 8’. Other parameters that influenced the ICER is given in the Fig 3A. In the single-stage screening, the parameters that had highest influence on the ICER were ‘3 monthly probability of CVD event from event free state of those in the CVD risk cohort 9’, ‘Three monthly probability of CHD event’ and ‘Three monthly probability of stroke from event free state in the CVD risk cohort 9’. The other factors that influenced the ICER is shown in the Fig 3B.

We assessed how the cost-effectiveness of the HEARTS-based intervention changes as its clinical effectiveness increases. We found that both screening strategies became more cost-effective as the intervention became more effective. When we considered the least and highest clinical effectiveness levels (10% and 80%), we found that the two-stage screening strategy was more cost-effective than the single-stage strategy. Specifically, the discounted ICER / QALY gained for two-stage screening ranged from 310 US$ (₹ 25,601) to ‐ 70 US$ (₹ -5,853), while the cost per QALY gained for single-stage screening ranged from 8,240 US$ (₹ 6,80,265) to 348 US$ (₹ 28,761) (Table 5).

**Table 5:** Scenario analysis of implementing two-stage and single-stage CVD risk assessment strategies in the district of Puducherry (N = 1).

Clinical effectiveness (%)	Two-stage screening	Single-stage screening
	ICER / QALY gained	ICER / QALY gained
	(discounted)	(discounted)
	US$ (₹)	US$ (₹)
10	310 (25,601)	8240 (6,80,265)
20	231 (19,114)	2046 (1,68,882)
40	105 (8,656)	1073 (88,588)
60	7 (581)	615 (50,777)
80	-70 (-5,853)	348 (28,761)

### The number of stroke and CHD cases

For a population of one lakh over a lifetime horizon of fifty years, the total number of stroke and CHD events estimated to occur in the two-stage and single-stage screening strategies were lesser compared to the no screening scenario. Among both the strategies, the two-stage screening was estimated to result in higher health gain compared to single-stage screening in terms of events that occurred (stroke and CHD) and cases averted (stroke and CHD) during the modeled period (Table 6).

**Table 6:** Total number of stroke and CHD events over fifty years among those aged ≥40 years and above in the district of Puducherry (N = 1,00,000).

Model outcomes	No screening strategy	*Two-stage screening	^#^Single-stage screening
Total Stroke events	8,178	5,972	4,190
Total CHD events	14,175	10,328	8,915
Stroke averted	-	2,207	3,988
CHD averted	-	3,848	5,261

*Population is screened initially using non-lab-based CVD risk assessment method, and those having ≥10% CVD risk were subjected to lab-based CVD risk assessment

^**#**^Population is screened only using the lab-based CVD risk assessment method

## Discussion

In the study, both the CVD screening strategies i.e. screening the population initially using non-lab-based CVD risk assessment method, and subjecting those having ≥10% CVD risk to lab-based CVD risk assessment (two-stage screening), and screening the population only using the lab-based CVD risk assessment method (single-stage screening) were cost-effective when compared to the current no screening scenario. Further, the two-stage screening was more cost effective than the single-stage screening strategy. However, total QALY gained, number of stroke and CHD events averted, and its associated deaths were estimated to be comparatively less in the two-stage screening than the single-stage screening. In comparison to no screening scenario, the model outcomes of both strategies were better.

In the study, both the two-stage and single-stage CVD screening strategies being cost-effective points out the cardiovascular health gains that could be harnessed if CVD screening strategies are implemented in the country. We also found that the two-staged screening was more cost-effective than the single-stage screening. This could be a favorable finding for LMICs like India as the total costs incurred for implementing lab-based screening were found to be comparatively costlier, as the whole population had to be subjected to diabetic and total cholesterol estimations. This finding supports the WHO recommendation of implementing non-lab-based screening as a precursor to lab-based screening in low-resource settings like India [7].

Evidence from developing countries also supports that stepwise CVD screening is more cost-effective than lab-only-based CVD risk screening [25]. Two main reasons could be attributed to this observation. First, non-lab-based assessment as an initial screening prevented most of the population with low CVD risk (<10%) from subjecting them to the costlier lab-based CVD screening. Second, it was evident from the singe stage screening that although the whole population was subjected to lab-based CVD risk estimation, there was only a marginal gain in terms of total QALYs gained compared to the two-stage screening strategy. This also indicates that the additional benefit in CVD risk prediction by subjecting the whole population to lab-based screening (testing for diabetes and total cholesterol) has only a marginally benefit over implementing the two-staged screening strategy causing two-stage screening as more cost-effective. A study conducted by the authors has also shown 89.4% concordance in CVD risk prediction of individuals in different CVD risk levels (<10, 10–19, and ≥20% total CVD risk) between the non-lab and lab-based CVD risk assessment indicating only a marginal benefit by subjecting the whole population to only the lab-based CVD risk assessment [15]. These finding also substantiates the use of non-lab-based screening for initial risk stratification of a population before employing lab-based screening.

In the study, we found that the total QALYs gained in the lab-based screening were comparatively higher than in the two-stage screening, although the increase was only marginal. Correspondingly, the total cases of stroke and CHD and its associated deaths were also estimated to be comparatively less in single-stage screening than in both the two-stage and no-screening scenarios. This observation supports the WHO recommendation of implementing the lab-based CVD assessment for the whole population when resources are available [7]. Substantiating this, evidence also showed that among those identified with ≥20% CVD risk using lab-based risk assessment, about 97% of them were having ≥10% risk by using the non-lab-based assessment indicating that implementing the lab-only-based risk assessment would be a better risk stratification approach compared to two-staged screening, for a population when resources are available [11]. 

For India, considering the higher costs involved and only a marginal gain in the total QALYs in the single-stage screening compared to two-stage screening, implementing lab-only based risk assessment at the national or regional level might be expensive. But, the single-stage (lab-based) screening might be cost-effective, especially when implemented among sub-groups of population with high prevalence of CVD risk factors, especially the total cholesterol, diabetes mellitus, and HTN.

Because, in such geographic settings with a high prevalence of biochemical risk factors, using the non-lab method as a preliminary screening for total CVD risk estimation would underestimate the CVD risk in the individuals, as non-lab-based method does not account for the risk contributed by diabetes and total cholesterol, and this would lead to high ‘false negatives’ for high total CVD risk. Substantiating this, a study has shown that among patients with diabetes having ≥20% CVD risk using lab-based assessment, only about 45% of men and 25% of women had ≥20% risk when screened using the non-lab method. Whereas, among those without diabetes with≥20% CVD risk using lab method, about 85% of men and 95% of women had ≥20% CVD risk using the non-lab method [11].

From the current study, it is also evident that rolling out CVD screening strategies implemented through community health workers is cost-effective. This finding supports several other studies conducted in LMICs that have implemented CVD screening using community health workers [25]. On the implementation front, as the current study has not assessed the budget impact of both CVD intervention strategies, future studies shall consider undertaking national/regional/state-specific budget impact analysis to present the affordability of interventions to the policy makers for consideration.

For implementing CVD screening strategies, there are several key factors that needs to be considered. The program managers need to forecast and strengthen the referral mechanisms and treatment requirements. Because, through a CVD screening program, population with raised blood pressure, raised blood glucose and hypercholesterolemia would be identified who needs to be initiated on treatment. Further, the public health system also needs to be equipped with resources for medical management of the population identified to have high total CVD risk. In India, where one-fourth and one tenth of the population have HTN and diabetes respectively, and with considerable disparities in addressing healthcare needs across states it is crucial that public health managers ensure the medical management of those identified through the survey [26, 27].

In this study, we utilized the most recent version of the WHO CVD risk prediction charts, to evaluate cost-effectiveness. The novelty of these charts has resulted in limited literature on this topic, making our study one of the earliest to assess the cost-effectiveness of the updated WHO CVD risk prediction charts in LMICs. Additionally, previous cost-effectiveness studies have employed diverse CVD risk screening tools and approaches from those used in our study, making it challenging to compare our findings with those of other studies [28–32].

India being an LMIC, we have presented the cost-effectiveness of both the CVD screening strategies advocated by the WHO. Therefore, the results obtained might hold for other LMICs. The CVD risk used for the study was estimated from an NCD risk factors survey conducted by the authors by following the WHO STEPS methodology in the district of Puducherry in India.

The study has certain limitations. In our Markov model, we assumed that an individual could experience either CHD or stroke, but not both. This assumption is because the likelihood of an individual who has had CHD developing a stroke (or vice versa) depends on various factors such as age, gender, lifestyle habits, and comorbid conditions like diabetes and hypertension [33], and including this possibility in the model would have added complexity and may have required additional assumptions to bridge data gaps. However, given the significant interplay between CHD and stroke events [34, 35], future studies could address this limitation by expanding the model to account for these possibilities.

It is important to acknowledge that the CVD risk estimates derived from prediction models may not always correlate well with actual data from longitudinal population studies. We agree that these predicted CVD risk estimates are the key determinants of the model outcomes on the total number of CVD events and associated management costs over a lifetime. However, the use of validated CVD risk prediction tools, such as the one advocated by the WHO for population-based CVD risk assessment and management for prevention and control of CVDs [7], would have minimized the bias on the study outcomes.

In the study, we used fixed values for utility scores for chronic conditions which might have an impact on the study outcomes. Incorporating of age-related deterioration of quality of life in the study cohort would have provided more accurate representation of the impact of the intervention on the quality of life over time especially among patients with comorbid chronic diseases. However, due to the lack of reliable data on age specific utility decrements for comorbid chronic diseases in the Indian population, we opted for using fixed values for utility scores. We acknowledge this limitation, and we suggest that the future studies in this area should consider incorporating age-specific utility decrements when reliable data are available.

The ICERs estimated for stepwise and singe stage screening were by assuming that the treatment effect of HEARTS intervention was 40%. Although several countries are in the implementation phase of the WHO HEARTS package, there is a lack of data on the effect of HEARTS intervention at a population level. To address this limitation, we have carried out scenario analysis by varying the intervention effect between 10% and 80%, all of which were cost-effective for both the screening strategies. For the model, we considered one-time population screening, whereas the WHO advocates for periodic screening to facilitate early detection and treatment initiation for CVD prevention.

In the study, we assumed that all those identified with raised blood pressure, diabetes mellitus, hypercholesterolemia, and those with different levels of CVD risk were initiated treatment as per HEARTS treatment guidelines. But, evidence suggests that the prevalence of screening, awareness, treatment initiation, and particularly treatment adherence varies across states and in sub-groups of populations [27]. Therefore, the assumption of cent percent on the screening coverage, treatment initiation, and treatment follow-up might impact the ICER estimated for the screening strategies. Further, studies have also shown that when screening coverage increases, it also increases the disutility more than the benefit of the intervention, as in the case of lab-based risk assessment where the majority population would be identified with low CVD risk (≤9) would undergo treatment which is usually long-term [36, 37]. In the study, the cost considered for stenting procedures was the unit cost. However, this cost could likely be a slight underestimate as the mean number of stents applied to a patient in case of cardiac / stroke events might be more than one. The budget impact analysis was not conducted in the current study, which could have informed the affordability of the screening strategies assessed.

## Conclusion

In India, compared to the current no screening scenario, implementing either of the CVD screening strategies, i.e., screening the population initially using a non-lab-based CVD risk assessment method, and subjecting those having ≥10% CVD risk to lab-based CVD risk assessment (two-stage screening) or screening the population only using the lab-based CVD risk assessment method (single-stage screening) would be cost-effective at an implementation effect of 40%. Future studies shall dwell on assessing the affordability of implementing the WHO HEARTS-based CVD strategies in Indian settings through budget impact analysis at different coverage levels of the screening.

## Supporting information

S1 File (ZIP)

## List of abbreviations

CVD: Caridovascular Disease
NCD: Non-Communicable Diseases
HTN: Hypertension
DM: Diabetes Mellitus
LMIC: Low And Middle-Income Countries
WHO: World Health Organization
CHD: Coronary Heart Disease
QALY: Quality Adjusted Life Years
ICER: Incremental Cost Effectiveness Ratio
OWSA: One-Way Sensitivity Analysis
NMB: Net Monitory Benefit
